# Supplementary material for: Integrating whole genome sequencing and machine learning for predicting antimicrobial resistance in critical pathogens: a systematic review of antimicrobial susceptibility tests
Source: PeerJ. 2024 Oct 9;12:e18213. doi: 10.7717/peerj.18213 (PMC11470768; doi:10.7717/peerj.18213)
Supplement: Supplemental Information 2 [file peerj-12-18213-s002.docx]

**Supplemental File Information:**

1. **Rationale for Conducting the Review:** The primary motivation for this systematic review is the urgent need to address the growing threat of antibiotic-resistant infections, which represent a significant burden on healthcare systems worldwide. By evaluating the effectiveness of machine learning models in predicting antimicrobial resistance using WGS and AST data, this review aims to provide insights into the potential of these technologies to enhance clinical decision-making and antibiotic stewardship.
2. **Contribution to Knowledge:** This systematic review makes a significant contribution to the existing body of knowledge by synthesizing and analyzing recent advancements in the application of machine learning for predicting AMR. This review uniquely integrates WGS and AST data types, offering a comprehensive overview of their combined utility in improving predictive accuracy. This work also highlights the top-performing ML models and identifies gaps in the current research, thereby guiding future studies and standardizing methodologies.
